# Whole-exome sequencing and gene-based rare variant association tests suggest that *PLA2G4E* might be a risk gene for panic disorder

**DOI:** 10.1038/s41398-017-0088-0

**Published:** 2018-02-02

**Authors:** Yoshiro Morimoto, Mihoko Shimada-Sugimoto, Takeshi Otowa, Shintaro Yoshida, Akira Kinoshita, Hiroyuki Mishima, Naohiro Yamaguchi, Takatoshi Mori, Akira Imamura, Hiroki Ozawa, Naohiro Kurotaki, Christiane Ziegler, Katharina Domschke, Jürgen Deckert, Tadashi Umekage, Mamoru Tochigi, Hisanobu Kaiya, Yuji Okazaki, Katsushi Tokunaga, Tsukasa Sasaki, Koh-ichiro Yoshiura, Shinji Ono

**Affiliations:** 1 Department of Neuropsychiatry, Unit of Translation Medicine, Nagasaki University Graduate School of Biomedical Sciences, Nagasaki, Japan; 2 Department of Human Genetics, Nagasaki University Graduate School of Biomedical Sciences, Nagasaki, Japan; 3 Department of Human Genetics, Graduate School of Medicine, The University of Tokyo, Tokyo, Japan; 4 Graduate School of Clinical Psychology, Professional Degree Program in Clinical Psychology, Teikyo Heisei University, Tokyo, Japan; 5 Arata-clinic, Nagasaki, Japan; 6 Department of Psychiatry, Psychosomatics, and Psychotherapy, Center of Mental Health, University of Würzburg, Würzburg, Germany; 7 Department of Psychiatry and Psychotherapy, Medical Center–University of Freiburg, Faculty of Medicine, University of Freiburg, Freiburg, Germany; 8 Division for Environment, Health and Safety, The University of Tokyo, Tokyo, Japan; 9 Department of Neuropsychiatry, Teikyo University School of Medicine, Tokyo, Japan; 10 Panic Disorder Research Center, Warakukai Med. Corp, Tokyo, Japan; 11 Department of Psychiatry, Koseikai Michino-o Hospital, Nagasaki, Japan; 12 Department of Physical and Health Education, Graduate School of Education, The University of Tokyo, Tokyo, Japan; 13 Aino-Ariake Hospital, Unzen, Nagasaki, Japan

## Abstract

Panic disorder (PD) is characterized by recurrent and unexpected panic attacks, subsequent anticipatory anxiety, and phobic avoidance. Recent epidemiological and genetic studies have revealed that genetic factors contribute to the pathogenesis of PD. We performed whole-exome sequencing on one Japanese family, including multiple patients with panic disorder, which identified seven rare protein-altering variants. We then screened these genes in a Japanese PD case–control group (384 sporadic PD patients and 571 controls), resulting in the detection of three novel single nucleotide variants as potential candidates for PD (chr15: 42631993, T>C in *GANC*; chr15: 42342861, G>T in *PLA2G4E*; chr20: 3641457, G>C in *GFRA4*). Statistical analyses of these three genes showed that *PLA2G4E* yielded the lowest *p* value in gene-based rare variant association tests by Efficient and Parallelizable Association Container Toolbox algorithms; however, the *p* value did not reach the significance threshold in the Japanese. Likewise, in a German case–control study (96 sporadic PD patients and 96 controls), *PLA2G4E* showed the lowest *p* value but again did not reach the significance threshold. In conclusion, we failed to find any significant variants or genes responsible for the development of PD. Nonetheless, our results still leave open the possibility that rare protein-altering variants in *PLA2G4E* contribute to the risk of PD, considering the function of this gene.

## Introduction

Panic disorder (PD) is characterized by recurrent and unexpected panic attacks, subsequent anticipatory anxiety, and phobic avoidance. The lifetime prevalence of PD is 4.7%, with a female preponderance^1^. Several genetic and epidemiological studies, including family and twin studies, have shown that genetic factors play an important role in the pathogenesis of PD. First-degree relatives of patients with PD have a sixfold greater risk for developing this disease^2^, and twin studies have shown a higher concordance rate in monozygotic twins than in dizygotic ones^3^. Neurodevelopmental disorders, including PD, are basically considered as multifactorial diseases. Thus far, several genome-wide association studies (GWAS) using DNA microarrays have focused on PD, but they have not been consistently replicated elsewhere^4–6^. Whole-exome sequencing (WES) is a powerful tool to detect genes causative of not only single-gene disorders, but also multifactorial ones, including autism spectrum disorder^7^. Regarding PD, to the best of our knowledge, only a few studies have explored genes causative of this condition using next-generation sequencing (NGS)^8–10^.

To identify genes related to PD, we performed WES in a Japanese family including several members with PD and identified variants linked to this disorder in this family. After identifying the genes that were candidates for causing PD in this family, the frequencies of all variants of these genes were compared between larger groups of PD cases and controls not related to this family. Here, we describe the results of NGS and the assessment of rare protein-altering variants of potential candidate genes in patients with PD and ethnically matched controls.

## Subjects and methods

### Clinical presentation of a Japanese family including multiple PD patients

**Fig. 1:**
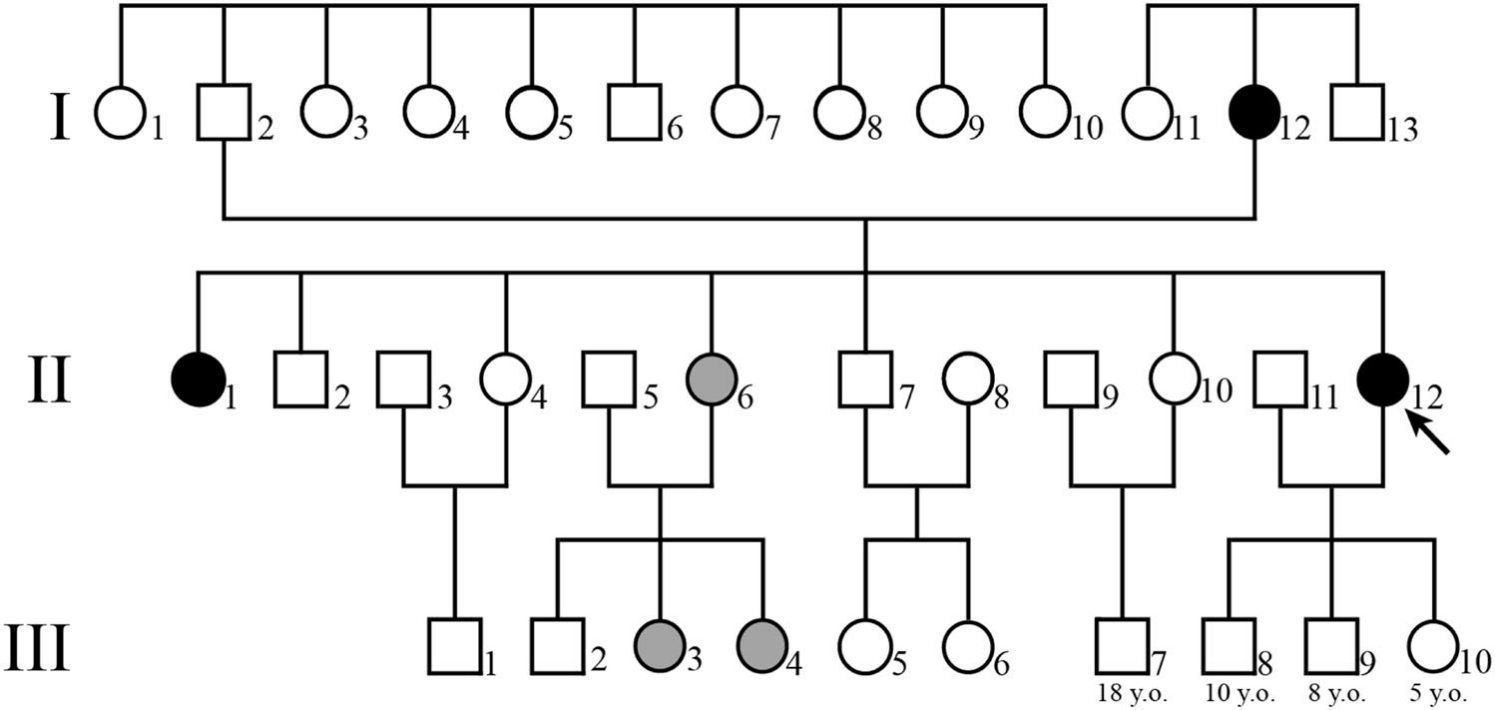
A Japanese family including multiple patients with panic disorder. The arrow indicates the proband. Black circles represent individuals (I-12, II-1, II-6, II-12, III-3, and III-4) diagnosed with panic disorder. Gray circles represent individuals (II-6, III-3, and III-4) diagnosed with panic disorder, who also showed schizophrenia-like symptoms, such as incongruity of emotional responses, social withdrawal, and lethargy. We performed whole-exome sequencing of four family members (I-2, I-12, II-10, and II-12) and direct sequencing of variants in nine family members (I-2, I-11, I-12, I-13, II-1, II-6, II-10, II-12, and III-4)

The proband (II-12) was a 40-year-old woman (Fig. 1). She had consulted a psychiatric clinic because of panic attacks and anticipatory anxiety. Her mother and elder sister (I-12 and II-1, respectively) had also consulted a psychiatric clinic because of symptoms similar to those in the proband. All of these three (I-12, II-1, and II-12) had been diagnosed with panic disorder by their attending psychiatrists. Among all affected individuals, three out of six (II-6, III-3, and III-4) had also consulted at a psychiatric hospital because of panic attacks and anticipatory anxiety and been diagnosed with panic disorder, as well as having incongruity of emotional responses, social withdrawal, and lethargy-like negative symptoms of schizophrenia. However, they did not fulfill the criteria for schizophrenia. All affected members in this family have similar panic symptoms in common, including panic attacks and anticipatory anxiety. We considered all of them as being “affected” and estimated that they have a shared disease-causing variant, and thus subjected them to further genetic analysis. Sertraline was effective for the treatment of their panic symptoms in many of the affected individuals. All subjects in this family were evaluated by multiple psychiatrists using the MINI International Neuropsychiatric Interview. All diagnoses of psychiatric diseases were made in accordance with the International Classification of Diseases, revision 10 (ICD-10), and the Diagnostic and Statistical Manual of Mental Disorders, fourth edition (DSM-IV). The father of the proband was the second eldest of ten siblings, who had not been suffering from any psychiatric diseases. The eldest sister and brother of the proband (II-1 and II-2, respectively) had never married and had no children. The second eldest sister and brother (II-4 and II-7) and their children (III-1, III-5, and III-6) had also not been suffering from any psychiatric diseases. Detailed information about them is summarized in Fig. 1 and Supplementary Table 1.

### Analysis of the mode of inheritance in this family using the SAGE program

To explore the mode of inheritance in this family, we performed complex segregation analysis using Statistical Analysis for Genetic Epidemiology, Release 6.4 (SAGE) (http://darwin.cwru.edu/sage/). Four of the family members (III-7, III-8, III-9, and III-10) were excluded from this analysis since they had not reached the mean age of the onset of PD (24 years old). We considered four different inheritance models: (a) sporadic model without familial residual association; (b) sporadic model with familial residual association; (c) Mendelian major gene models with autosomal-dominant inheritance; and (d) Mendelian major gene models with autosomal recessive inheritance. Akaike’s Information Criterion (AIC) was used to estimate the best fitting model among these four alternatives.

### Japanese and German PD case–control groups

A total of 669 sporadic PD cases and 859 control samples were collected from two countries, Japan and Germany (952 Japanese and 576 German). All of these patients with PD had been diagnosed by psychiatrists in accordance with ICD-10, DSM-III, or DSM-IV. Their details are summarized in Supplementary Table 2. All participants provided informed consent to undergo genetic research, which was approved by the ethics committees.

### DNA extraction

We recruited nine members of this family for this study, namely, five affected and four unaffected individuals (Fig. 1). Genomic DNA was extracted from peripheral blood leukocytes using a QIAamp DNA Midi kit (QIAGEN, Hilden, Germany).

### Whole-exome sequencing

We assessed DNA quality using NanoDrop 2000 (Thermo Fisher Scientific, Waltham, MA, USA) and Qbit Fluorometer Nucleic Acid Quantification (QIAGEN, Hilden, Germany) and selected four high-quality DNA samples (I-2, I-12, II-10, and II-12) for WES. We performed WES on two affected individuals with PD and two unaffected individuals in this family (Fig. 1), using Agilent SureSelect Exome Target Enrichment System v5 (Agilent Technologies, Santa Clara, CA, USA), followed by paired-end sequencing on SOLiD 5500xl sequencers (Life Technologies, Carlsbad, CA, USA). Mean depth and coverage rate of all samples are summarized in Supplementary Table 3.

### Sequencing data analysis

Fastq format files were generated by bcl2fastq software. Novoalign software (Novocraft Technologies, Kuala Lumpur, Malaysia) was used to align reads on the hg19/GRCh37 human reference genome sequence. Aligned reads were sorted by Novosort software (Novocraft) and subjected to the marking of PCR and optical duplication by MarkDuplicates in the Picard tools package (http://broadinstitute.github.io/picard/). The Genome Analysis Toolkit (GATK)^11^ was used to perform local realignment (GATK IndelRealigner) and variant call (GATK HaplotypeCaller) was implemented in an in-house workflow management tool^12^. Single nucleotide variations (SNVs) and insertions/deletions (indels) were annotated using ANNOVAR software^13^. Substitutions that met the following criteria were selected as “deleterious”: (1) mutations: lead stop gain, stop loss, nonsynonymous, or splice site mutations according to GENCODE basic version 19 downloaded from the UCSC genome browser; (2) alternative allele frequencies at mutation loci ≤0.5% in the following databases: 1000 Genomes Project data on all populations released in October 2014^14^; NIH NHLBI 6515 exome data (http://evs.gs.washington.edu/EVS/); Exome Aggregation Consortium (ExAC) 65000 exome data^15^; Human Genomic Variation Database (HGVD) exome data of 1208 individuals collected in Japan^16^; SNV allele frequency collected from whole-genome sequencing data of 2049 healthy Japanese individuals (2KJPN) (https://ijgvd.megabank.tohoku.ac.jp); and (3) mutations not included in the UCSC segmental duplication region.

### Sanger sequencing

We performed Sanger sequencing to confirm 127 variants shared among two affected individuals (II-1 and II-12) but absent from an unaffected one (I-2), which emerged from the WES. All primers were designed using Primer3^17^. Genomic DNA (5 ng) was amplified using KOD FX Neo polymerase (Toyobo, Osaka, Japan) by PCR in 20-μl reactions. PCR mixtures were subjected to the following thermal cycling conditions: initial denaturation at 94 °C for 2 min; 32 cycles of denaturation at 98 °C for 10 s, annealing at 60 °C for 15 s, and extension at 68 °C for 30 s; and then final extension at 68 °C for 5 min. The PCR products were purified with AMPureXP^®^ (Agencourt, Beverly, MA, USA) and sequencing reactions were performed using the BigDye Terminator Cycle Sequencing Kit v3.1 (Applied Biosystems, Foster City, CA, USA), in accordance with the manufacturer’s instructions. After purification using CleanSEQ (Agencourt), the PCR products were separated and run on an ABI Genetic Analyzer 3130 (Applied Biosystems) and the electropherograms were evaluated using 4Peaks software (http://nucleobytes.com/4peaks/). Ten SNVs/indels were detected in all five affected family members. The original ten variants are summarized in Table 1.

**Table 1:** Nine SNVs and one deletion were found in all affected members

					Genotypes									Penetrance	LOD score
Gene	Variants	Protein change	dbSNP	HGVD	I-12	II-1	II-6	II-12	III-4	I-2	I-11	I-13	II-10		
*ADRA1D*	chr20: 4202670; G>A	p.Arg407Cys	None	0.0019	G/A	G/A	G/A	G/A	G/A	G/G	G/G	G/G	G/A	0.83	0.827847
*ATP8B4*	chr15: 50271864; C>T	p.Trp328X	rs142863209	0.0032	C/T	C/T	C/T	C/T	C/T	C/C	C/T	C/T	C/C	0.71	0.417835
*C10orf120*	chr10: 124459278; T>C	p.Gln10Arg	None	0.0014	T/C	T/C	T/C	T/C	T/C	T/T	T/C	T/T	T/T	0.83	0.828729
*FAM82A2*	chr15: 41044217; C>T	p.Arg116Gln	rs200285306	0.0074	C/T	C/T	C/T	C/T	C/T	C/C	C/T	C/T	C/C	0.71	0.445693
*GALNTL5*	chr7: 151711868; A>T	p.Asp389Val	rs199860896	0.0009	A/T	A/T	A/T	A/T	A/T	A/A	A/T	A/T	A/A	0.71	0.401112
*GANC*	chr15: 42631993; T>C	p.Leu657Pro	None	None	T/C	T/C	T/C	T/C	T/C	T/T	T/C	T/C	T/T	0.71	0.395025
*GFRA4*	chr20: 3641457; G>C	p.Arg176Gly	None	None	G/C	G/C	G/C	G/C	G/C	G/G	G/G	G/G	G/C	0.83	0.829517
*HDHD3*	chr9: 116136256; G>-	p.Arg127fs	None	0.0058	G/-	G/-	G/-	G/-	G/-	G/G	G/-	G/-	G/-	0.63	0.114774
*KIAA0319*	chr6: 24582481; G>C	p.Ser351Cys	None	0.0036	G/C	G/C	G/C	G/C	G/C	G/G	G/C	G/C	G/C	0.63	0.106594
*PLA2G4E*	chr15: 42342861; G>T	p.Thr14Asn	None	None	G/T	G/T	G/T	G/T	G/T	G/G	G/T	G/T	G/G	0.71	0.395025

The parameter of the penetrance ratio was varied depending on each phenotype/genotype rate in this family. The The LOD score of two-point linkage analysis were calculated using FASTLINK program.

### Linkage and haplotype analysis of candidate variants

We performed linkage analysis of ten potential candidate variants using the MLINK program of the LINKAGE/FASTLINK software package (https://www.ncbi.nlm.nih.gov/CBBresearch/Schaffer/fastlink.html) assuming a dominant model. The parameter of the penetrance ratio was varied depending on each phenotype/genotype rate in this family (Table 1).

### Mutational analysis of all exons among seven genes

We sequenced all exons of the seven genes (*ADRA1D*, *ATP8B4*, *C10orf120*, *GANC*, *GFRA4*, *HDHD3*, and *PLA2G4E*) identified by the above procedure in 573 samples (381 Japanese patients with PD; 96 German patients with PD; 96 German controls) by amplicon sequencing. WES in-house data on 571 Japanese individuals from another project were also used. Primers were designed using Ion Ampliseq Designer (https://www.ampliseq.com/browse.action) (Supplementary Table 4). In amplicon sequencing, all exons within these seven genes were amplified using KAPA2G Multiplex PCR Kit (KAPA Biosystems, Woburn, MA, USA) or KOD Extreme HotStart Polymerase (Toyobo, Osaka, Japan) by PCR. The samples were subjected to the following thermal conditions: initial denaturation at 95 °C for 2 min; and then 25 cycles of denaturation at 95 °C for 30 s, annealing at 60 °C for 120 s, and extension at 72 °C for 120 s. PCR amplicons were sonicated to a size of 180 bp using Covaris E220 (Covaris, Woburn, MA, USA). End-repair, A-tailing, and adapter ligation were performed in accordance with the manufacturer’s protocol for KAPA HTP Library Preparation Kits for Illumina Platforms. After adapter ligation, the samples were amplified using KAPA HiFi HotStart Ready Mix (KAPA Biosystems, Wilmington, MA, USA) by PCR with indexed primers. Samples were subjected to the following thermal cycling conditions: initial denaturation at 98 °C for 45 s; seven cycles of denaturation at 98 °C for 15 s, annealing at 60 °C for 30 s, and extension at 72 °C for 30 s; and final extension at 72 °C for 1 min. Prepared amplicon libraries were sequenced using HiSeq 2500 (Illumina). The mean depth of all samples is summarized in Supplementary Table 5.

### Single-variant association test

To detect the single variants associated with PD, we used PLINK^18^ to estimate *p* value and odds ratios. In this study, we filtered out the variants using the following criteria: alternative allele frequencies of less than 0.005 in the samples in this study, exact test of Hardy–Weinberg equilibrium: *p* < 1 × 10^−6^, and call rates of less than 90%. In total, 16 and 19 variants were found in the Japanese and Germans, respectively, so the significant thresholds of *p* values were set to 0.0031 in the Japanese and 0.0026 in the Germans, in accordance with Bonferroni correction.

### Gene-based rare variant association tests

To test differences in the mutational burden of the candidate genes, we used Efficient and Parallelizable Association Container Toolbox (EPACTS). In the EPACTS algorithms, we used three different analyses (CMC^19^, Madsen–Browning^20^, and SKAT-O^21^). We removed common variants using the following criteria: alternative allele frequency of more than 0.005 in any public database (ExAC, ESP6500, 1000 Genomes, HGVD, and ToMMo 2KJPN) or more than 0.005 in control samples in this study; call rate of less than 90% in the study samples; and genotyping quality score of less than 99. In German samples, *GFRA4* was removed from the analysis because of the lack of a variant allele. The significance threshold here was set to 0.0167, in accordance with Bonferroni correction.

## Results

### Segregation analysis

The segregation analysis using SAGE algorithms showed that the “Mendelian major gene models with autosomal-dominant inheritance,” which was the plausible mode of inheritance in this family. This result indicated that this family carries a single locus with a strong impact on PD. The AIC scores are summarized in Supplementary Table 6.

### Linkage analysis

Two-point logarithm of the odd (LOD) scores were calculated by FASTLINK software. The LOD scores of ten variants are summarized in Table 1.

### Whole-exome sequencing and Sanger sequencing

We identified 127 functionally significant variants shared by the two affected individuals subjected to WES. To identify compelling candidates, we searched variants shared among all affected members by Sanger sequencing of these 127 variants (Fig. 1). As a result, nine SNVs and one deletion were found in all affected members (Table 1).

### Mutational screening of seven familial substitutions in Japanese and German PD case–control groups

To examine the allele frequencies of these candidate alterations, we screened these substitutions in Japanese and Germans with or without PD (Supplementary Table 2). In the Japanese (384 patients with PD and 571 matched controls), we found some SNVs that had been identified in the WES (chr20: 4202670, G>A in *ADRA1D*; chr15: 50271864, C>T in *ATP8B4*; chr10: 124459278, T>C in *C10orf12*0; chr9: 116136256, G>- in *HDHD3*). These four variants were also observed in controls. Furthermore, none of these variants showed a significant preponderance in PD cases relative to the level in controls, so they were considered to be polymorphisms that did not have a major role in the development of PD. However, three SNVs (chr15: 42631993, T>C in *GANC*; chr15: 42342861, G>T in *PLA2G4E*; chr20: 3641457, G>C in *GFRA4*) were “novel variants,” since they were not observed in controls or in any databases and their allele frequencies and estimated pathogenic impact could not be evaluated (Table 2). Taking these findings together, we considered these three SNVs to be potential pathogenic variants in this family (Table 2). In contrast, in the Germans (288 patients and 288 controls), none of these seven variants was found in either patients or controls.

**Table 2:** Allele counts of familial SNVs/Indels in Japanese PD case–control cohorts

Gene	Variants	Mutation allele counts		Allele frequency			*P* value	OR
		PD (*N* = 957)	Control (*N* = 1718)	HGVD	ExAC03	ESP6500		
*ADRA1D*	chr20: 4202670; G>A	0	1	0.0019	0.0002	0.00313	1	0.5
*ATP8B4*	chr15: 50271864; C>T	2	3	0.0032	0.0002	0.003173	1	1
*C10orf120*	chr10: 124459278; T>C	2	2	0.0014	1.68E−05	0.00187	1	1.5
*GANC*	chr15: 42631993; T>C	0	0	None	None	None	NA	NA
*GFRA4*	chr20: 3641457; G>C	0	0	None	None	None	NA	NA
*HDHD3*	chr9: 116136256; G>-	1	3	0.0005	8.25E−06	0.001166	0.6539	0.5
*PLA2G4E*	chr15: 42342861; G>T	0	0	None	None	None	NA	NA

*P* values and odds ratio were estimated by Fisher’s exact test

### Single-variant association test

To identify unique loci associated with PD in the three candidate genes containing the SNVs mentioned above (*GANC*, *GFRA4*, and *PLA2G4E*), we tested 27 SNVs identified by amplicon sequencing using PLINK. The results showed that no single loci were significantly associated with PD in both Japanese and Germans (Table 3 and Supplementary Tables 7 and 8).

**Table 3:** Results of single-variant association tests

	Variants	Mutation allele counts		*P* value	OR
Japan gene		PD (*N* = 762)	Control (*N* = 1142)		
* PLA2G4E*	chr15: 42298189; C>T	12	7	0.06299	2.434
* GFRA4*	chr20: 3640641; A>G	12	8	0.069	2.3
* GANC*	chr15: 42643538; G>A	53	60	0.1659	1.329
* GANC*	chr15: 42579984; A>G	52	60	0.197	1.299
* PLA2G4E*	chr15: 42292389; A>G	2	8	0.2098	0.3507
Germany gene		PD (*N* = 192)	Control (*N* = 192)		
* GANC*	chr15: 42619508; C>T	15	25	0.09344	0.539
* GFRA4*	chr20: 3640823; C>T	96	75	0.0971	1.427
* GFRA4*	chr20: 3644082; T>C	1	5	0.1168	0.1895
* GANC*	chr15: 42579984; A>G	17	9	0.1543	1.933
* GANC*	chr15: 42631928; C>T	17	24	0.1892	0.6387

Top five loci are displayed with the lowest *p* value first. The significance thresholds were as follows: Japan, 0.0031; and Germany, 0.0026

### Gene-based rare variant association tests

To examine whether there was an excess of rare mutations resulting in protein alterations of three genes (*GANC*, *GFRA4*, and *PLA2G4E*) in patients with PD compared with their levels in controls, we performed gene-based rare variant association tests using EPACTS algorithms. In the Japanese group (384 patients and 571 controls), *PLA2G4E* showed the lowest *p* values (*p* = 0.0715 in CMC, *p* = 0.0658 in Madsen–Browning, *p* = 0.1624 in SKAT-O), but did not reach the significance threshold. *PLA2G4E* also showed the lowest *p* values (*p* = 0.0652 in CMC, *p* = 0.0347 in Madsen–Browning, *p* = 0.0646 in SKAT-O) in the German population (Table 4 and Supplementary Tables 9 and 10).

**Table 4:** Results of gene-based rare variant association tests

	Number of markers	*P* value CMC	Madsen–Browing	SKAT-O
Japanese gene				
* GANC*	6	0.9977	0.9939	0.5172
* GFRA4*	3	0.9744	0.1026	0.1699
* PLA2G4E*	12	0.0715	0.0658	0.1624
Germany gene				
* GANC*	5	0.2751	0.2645	0.415
* PLA2G4E*	6	0.0652	0.0347	0.0646

The significance threshold was set to 0.0167

## Discussion

In this study, we focused on rare protein-altering variants in a Japanese family, including multiple patients, with PD to identify genetic variants contributing to the risk of this disorder. Recent studies have indicated that the genetic risk of psychiatric disorders involves the combined effects of many common variants of small effect, as well as rare variants of large effect including gene-disrupting mutations that might have a strong impact on the development of diseases^22–24^. They have also suggested the possibility that an extremely large effect of even just a single variant could cause diseases. In the present study, we hypothesized that there may be rare highly penetrant mutations with a very large effect in this family; this was supported by the findings of affected individuals through three generations and the autosomal-dominant inheritance mode identified from the segregation analysis. Assuming that the penetrance of the rare mutation was going to be high with incomplete penetrance, there is a possibility that the siblings of the proband were inherited the rare mutation with a large effect. First, for primary candidates, we selected variants that all affected individuals carried but that the father of the proband did not carry from the results of both WES and Sanger sequencing, resulting in ten variants remaining. The LOD score of these ten candidates was in the range of 0.11–0.83. Given the small family size here, it is unlikely that a LOD score of 3 would be achieved^25^. The LOD score >0 obtained here should not be overlooked in this particular case of a single small family. Next, we filtered out three of these ten variants since three SNVs that had already been registered in the dbSNP database (https://www.ncbi.nlm.nih.gov/SNP/) or showed a high allele frequency (>0.003) in the HGVD database; however, we included a nonsense mutation in *ATP8B* and a one-base deletion in *HDHD3* are included due to them having a high probability of being deleterious mutations (Table 1). Thus, we selected seven variants as being potentially pathogenic in this family. Subsequently, we eliminated four of these seven candidate variants since none of them showed significant accumulations in PD from the results of sequencing of our case–control group, despite each allele frequency being less than 0.003 in a public database. Seven variants were thus eliminated from the primary ten candidates because they were assumed to have very low penetrance. Recent studies have also suggested that minor allele frequencies have a strong association with their odds ratios^22,23^, which supported our filtering strategy. As a result of this filtering, we identified three rare protein-altering variants as potential disease-causing candidates after resequencing and filtering. We then performed mutational burden analyses of these three potential candidate genes. A single-variant association test of these three potential candidates did not show any association with PD. Because association testing using common single variants is effective for detecting loci with small phenotypic effects^26^; GWAS is a suitable method to detect common loci associated with complex diseases including psychiatric conditions^27,28^. GWAS focusing on PD has already been reported in the Japanese and German populations, which identified several candidate genes (e.g., *TMEM132D*, *PKP1*, *PLEKHG1*, and *CLU*)^6,29^, although these do not overlap with the three candidate genes identified in the present study. In gene-based rare variant association tests, *PLA2G4E* showed the lowest *p* value among the three candidates in this study, but did not reach the significance threshold in either the Japanese or the German group. There are several possible explanations for this. First, the significance threshold after Bonferroni correction might be too stringent to achieve the precise detection of meaningful variants in gene-based rare variant methods. Recent studies have indicated that existing gene-based rare variant methods might not be able to detect disease-related variants at the exome-wide significance threshold despite the effect size of variants being relatively large^30–33^. They also suggested that the absence of a positive signal in gene-based association studies does not exclude a role of rare variation in diseases. The second possibility is that the effect size of variants is not so large. Moutsianas et al. demonstrated that the power of detection in gene-based association tests is inversely related to the degree of linkage disequilibrium between causal variants at a locus^30^. The effect size of three potential candidates might have influenced to our negative results. The last possible explanation is that the sample size was too small. To boost the statistical power of the gene-based rare variant association method, it is usually necessary to increase the sample size^30–32^. A small sample size is thus one of the limitations of our study.

To the best of our knowledge, no study has yet reported an association between *PLA2G4E* and PD. *PLA2G4E* is very poorly characterized, although a few studies have revealed its functions. For example, Ohto et al. demonstrated that recombinant PLA2G4E influences phospholipase activity^34^, while Ogura et al. showed that it is strongly expressed in the brain, especially in neurons, and regulates bioactive lipids, namely, *N*-acyl phosphatidylethanolamines and *N*-acyl ethanolamines including the endocannabinoid anandamide, by mobilizing intracellular calcium in mammalian cells^35^. They also surmised that the PLA2G4E-regulated pathway might provide new therapeutic strategies for neuropsychiatric diseases. Interestingly, in recent years, a number of studies have presented evidence for the involvement of the endocannabinoid system (ECS) in PD^36,37^. The cannabinoid type 1 receptors influence the glutamatergic and GABAergic regulation of anxiety and fear response, which can be recognized as a key regulatory element in the pathogenesis of PD^38^. However, functional analyses of variants of *PLA2G4E* have yet to be performed, which are needed to draw definitive conclusions on the causal association between these variants in *PLA2G4E* and ECS/neurotransmitter release.

In conclusion, we failed to identify any genes responsible for the development of PD through exome sequencing in a Japanese family featuring multiple patients with PD as well as through a case–control study. *PLA2G4E* showed the lowest *p* value among the three candidates in this study in both of the two different ethnic groups; however, none of the genes or variants reached the threshold for statistical significance. Nonetheless, considering both our genetic findings and protein functions, the possibility remains that *PLA2G4E* could be involved in the pathogenesis of PD and become a new therapeutic target for this disorder.

## Electronic supplementary material

Supplementary Table 1

Supplementary Table 2

Supplementary Table 3

Supplementary Table 4

Supplementary Table 5

Supplementary Table 6

Supplementary Table 7

Supplementary Table 8

Supplementary Table 9

Supplementary Table 10
